# Histological evaluation of pulpal healing following direct pulp capping with polytetrafluoroethylene or melatonin, with or without adjunctive low-level laser therapy in Wistar rat molars

**DOI:** 10.1038/s41598-026-64337-7

**Published:** 2026-08-30

**Authors:** Samah Ahmed Abdelaziz, Latifa Mohamed Abdelgawad, Iman A. Fathy, Sayed eltayab, Ali Mohamed Saafan

**Affiliations:** 1 Endodontics Ain Shams University, Cairo, Egypt; 2 Medical Applications of Lasers Department, National Institute of Laser Enhanced Sciences (NILES), Cairo University, Giza, Egypt; 3 Oral Biology Department, Faculty of Dentistry, Ain Shams University, Cairo, Egypt

**Keywords:** Melatonin, Low-level laser therapy, Dental pulp capping, Dentin bridge formation, Wistar rats, Cell biology, Diseases, Medical research, Stem cells

## Abstract

This study evaluated pulpal healing following direct pulp capping with polytetrafluoroethylene (PTFE) or melatonin (MEL), with or without adjunctive low-level laser therapy (LLLT), in Wistar rat molars. Fifty-two maxillary first molars from 26 male Wistar rats were included. Forty-eight molars with standardized pulp exposures were randomly allocated to four groups (*n* = 12): Group I, PTFE; Group II, MEL; Group III, PTFE + LLLT; and Group IV, MEL + LLLT; four unexposed molars served as negative controls. LLLT (650 nm, 100 mW; fluence 11.9 J/cm^2^) was applied after placement of the capping material. Irradiation was performed on alternate days, with animals receiving either three sessions (1-week group) or six sessions (2- and 4-week groups). Histological evaluation was performed after 1, 2, and 4 weeks, assessing inflammation, necrosis, vascularity, fibrosis, odontoblast-like layer organization, dentin bridge formation, and dentin bridge thickness. LLLT-treated groups showed reduced inflammation, improved odontoblast-like layer organization, enhanced vascularity, and greater dentin bridge thickness compared with non-irradiated groups. The MEL + LLLT group demonstrated the most favorable overall histological profile. Adjunctive LLLT was associated with improved pulpal healing outcomes, while melatonin showed supportive effects on the pulpal microenvironment. Their combined application produced the most favorable histological outcomes and warrants further investigation in vital pulp therapy.

## Introduction

 Preserving dental pulp vitality and functionality is a fundamental objective in conservative dentistry and regenerative endodontics. Direct pulp-capping (DPC) is a minimally invasive procedure that aims to maintain dental pulp health after exposure by protecting the tissue, stimulating reparative dentinogenesis, and supporting long-term tooth function^1^.The success of DPC depends greatly on the biological properties of the applied capping material, particularly its biocompatibility, anti-inflammatory activity, and ability to induce odontoblastic differentiation^2,3^.

Melatonin (N-acetyl-5-methoxy-tryptamine), an indoleamine hormone primarily secreted by the pineal gland, has attracted growing interest as a biologically active adjunct capable of modulating oxidative and inflammatory responses relevant to regenerative pulp therapies. Beyond its cytoprotective actions, melatonin supports cellular viability by stabilizing mitochondrial redox balance, mitigating oxidative stress, and promoting osteogenic and odontogenic differentiation in dental-derived stem cells^4–7^. Preclinical studies further demonstrate that melatonin enhances mineralized tissue deposition, maintains odontoblastic organization, and attenuates pulpal inflammation, reinforcing its potential role in vital pulp therapy^8^.

Low-level laser therapy (LLLT), also known as photobiomodulation, represents an important biophysical modality in regenerative dentistry through its ability to modulate cellular bioenergetics. Irradiation in the red to near-infrared spectrum enhances mitochondrial respiratory activity and ATP synthesis, thereby promoting cellular proliferation, angiogenesis, and collagen deposition^9,10^. Within dental pulp tissue, LLLT has been shown to stimulate dental pulp stem cell proliferation, regulate inflammatory mediator expression, and accelerate dentin bridge formation^9,11,12^.

Mechanistic investigations indicate that LLLT influences redox-sensitive and MAPK-related pathways at the mitochondria, fostering a pro-healing microenvironment when applied under well-controlled irradiation parameters^9–11^. These biological responses exhibit a dose-dependent relationship, emphasizing the need for standardized laser protocols to achieve predictable therapeutic outcomes^10,13^.

Although melatonin and LLLT independently demonstrate considerable regenerative potential, to our knowledge, few studies have investigated whether LLLT-induced mitochondrial signaling can enhance or complement melatonin in vital pulp therapy. Their mechanisms are inherently complementary: melatonin mitigates oxidative and inflammatory stressors that may limit cellular responsiveness, while LLLT augments mitochondrial metabolism, vascular perfusion, and proliferative activity. This coordinated modulation of biochemical and bioenergetic pathways provides a strong theoretical basis for exploring synergistic enhancement of pulpal healing^9–12,14^. Recent advancements further reinforce this rationale, including mechanistic insights into LLLT signaling^15^, evidence of angiogenesis and tissue repair^16^, melatonin’s MAPK-mediated osteogenic effects^17^, and optimized LLLT parameters for fibroblast proliferation^18^.

Rat maxillary molars offer a well-established translational model for studying pulp biology due to their comparable tissue architecture, predictable healing dynamics, and suitability for controlled experimental manipulation. Therefore, the present study aimed to evaluate the histological response of Wistar rat dental pulp to melatonin as a direct pulp-capping agent, both alone and combined with low-level laser therapy, using polytetrafluoroethylene (PTFE) thread seal tape as an inert experimental control. The alternative hypothesis posited that melatonin-mediated redox modulation would enhance LLLT-induced photobiostimulatory effects, resulting in improved pulpal healing outcomes—including inflammation resolution, odontoblastic differentiation, and hard tissue formation—compared with either treatment alone or the control.

## Materials and methods

### Ethical approval

All experimental procedures were approved by the Institutional Animal Care and Use Committee (IACUC) of Cairo University, Egypt (Approval No. CUIF2321), and were conducted in accordance with institutional and international guidelines for the care and use of laboratory animals. The study adhered to the Animal Research: Reporting of In Vivo Experiments (ARRIVE 2.0) guidelines^19^ and complied with European Directive 2010/63/EU on the protection of animals used for scientific purposes^20^. Every effort was made to minimize the number of animals used and to alleviate pain and distress throughout the experimental procedures.

### Selection of animals and sample size

Sample size was determined a priori using G*Power software (version 3.1; Heinrich Heine University, Düsseldorf, Germany). The calculation was based on a one-way analysis of variance (fixed effects, omnibus), using an effect size of 0.64 derived from differences in inflammatory response scores reported by Islam et al.^21^, with a significance level (α) of 0.05 and a statistical power (1 − β) of 0.95. The minimum required sample size was estimated as 48 experimental teeth obtained from 24 animals, equally allocated among the four experimental groups (*n* = 12 teeth per group). Four additional teeth from two animals were included as untreated negative controls for baseline histological comparison and were excluded from the statistical analysis, resulting in a total of 52 maxillary first molars obtained from 26 rats.

Twenty-six healthy male Wistar rats aged 6–10 weeks and weighing 210–250 g were included in the study. Animals were housed in groups of four per cage under standardized environmental conditions (22 ± 1 °C, 50–55% relative humidity, 12-hour light/dark cycle) with ad libitum access to a standard laboratory diet and water.

Because the intervention and all histological outcome measures were localized to the treated pulp tissue, the tooth was considered the experimental unit. This approach is consistent with previous preclinical direct pulp-capping studies using multiple teeth per animal^21–24^.

### Study design

The study evaluated pulpal healing following direct pulp capping using polytetrafluoroethylene (PTFE) or melatonin, with or without adjunctive low-level laser therapy (LLLT). Four experimental groups were investigated.

#### Group I (PTFE)

The exposed pulp of the maxillary first molar was directly capped with polytetrafluoroethylene (PTFE).

#### Group II (MEL)

The exposed pulp of the maxillary first molar was directly capped with melatonin.

**Group III (PTFE + LLLT)**: The exposed pulp of the maxillary first molar was directly capped with PTFE and subsequently irradiated with low-level laser therapy using a 650-nm diode laser operating at 100 mW in continuous-wave mode. Laser irradiation was delivered for 30 s per cycle, repeated for two consecutive cycles (total irradiation time: 1 min) per session every other day, corresponding to a fluence of 11.9 J/cm^2^.

#### Group IV (MEL + LLLT)

The exposed pulp of the maxillary first molar was directly capped with melatonin and subsequently irradiated with low-level laser therapy using the same irradiation parameters as those described for Group III.

Histological evaluation was performed at 1, 2, and 4 weeks after treatment. Animals were euthanized at the respective experimental endpoints.

### Randomization and blinding

Animals were randomly allocated to either the laser-treated or non-laser treatment groups using a computer-generated randomization sequence. Allocation concealment was achieved using sealed opaque envelopes. Because laser irradiation was delivered to both maxillary first molars of each animal, allocation to laser treatment was performed at the animal level.

Within each animal, allocation of the pulp-capping materials (polytetrafluoroethylene [PTFE] or melatonin) to the right or left maxillary first molar was independently randomized using a computer-generated randomization sequence.

All animals were managed under identical experimental conditions throughout the study. All operative procedures were performed by the same operator using a standardized protocol for anesthesia, cavity preparation, pulp exposure, material application, laser irradiation, postoperative care, and animal handling. Because laser irradiation had to be delivered by the operator, operator blinding was not feasible. However, the standardized protocol was applied consistently to all experimental groups to minimize performance bias.

Histological evaluation was performed independently by two examiners who were blinded to treatment allocation, in accordance with the ARRIVE 2.0 recommendations^19^. Any discrepancies between examiners were resolved by consensus.

### Anesthesia

General anesthesia was induced by intramuscular administration of ketamine (80–100 mg/kg) and xylazine (5–10 mg/kg) according to the protocol described by Barriga-Rivera et al.^25^. Adequate anesthetic depth was confirmed by the absence of corneal and pedal withdrawal reflexes before the procedure. Mouth opening was maintained using a mouth gag secured to the maxillary and mandibular incisors to facilitate operative access. Local infiltration anesthesia with 2% lidocaine containing 1:100,000 epinephrine was administered adjacent to the operative site to provide local analgesia, reduce intraoperative bleeding, and standardize operative conditions during pulp exposure and direct pulp capping.

### Cavity preparation and pulp exposure

Occlusal surfaces were disinfected with 0.12% chlorhexidine solution. Standardized Class I cavities were prepared using a sterile low-speed round carbide bur (#1/2, head diameter 0.6 mm; CA shank, MANI Inc., Tochigi, Japan) mounted on a low-speed contra-angle hand piece driven by an NSK electric micromotor operating at 20,000 rpm under continuous saline irrigation. To maintain cutting efficiency and ensure procedural standardization, each bur was used for a maximum of two molar preparations. Cavity depth was standardized to approximately 0.5 mm. Pulp exposure was confirmed by the presence of visible bleeding. Hemostasis was achieved using sterile saline-moistened paper points without the use of chemical hemostatic agents, in accordance with previously established direct pulp-capping protocols^21,26^.

### Application of different treatment modalities for direct pulp capping in the experimental groups

**Melatonin** Two orodispersible sheets containing 3 mg of Melatonin (each sheet contains 1.5 mg) (Melacryst^®^, Ora-dispersible films, Crystal pharma, 6th industrial zone, 6th of October, Giza, Egypt)^27^.

**Laser device and irradiation parameters** LLLT was delivered using a 650 nm diode laser device (Woodpecker^®^, China) operating in continuous wave mode. Output power was set at 100 mW. A biostimulation hand piece with an 8.0 mm diameter tip was used, producing a spot area of 0.503 cm^2^^27^.

In Groups I and III (PTFE and PTFE + LLLT), polytetrafluoroethylene (PTFE) thread seal tape (RS Thread Seal Tape, RS Components Ltd., Corby, UK) was sterilized by autoclaving before use, aseptically trimmed, and adapted directly over the pulp exposure to serve as an inert experimental capping material for comparison with biologically active pulp-capping agents^28–31^. In Groups II and IV (MEL and MEL + LLLT), melatonin was aseptically adapted directly over the exposure site.

### Final restoration

All cavities were immediately restored with conventional glass ionomer cement (Medifill^®^; Promedica, Germany) according to the manufacturer’s instructions and established pulp-capping protocols^32^.

### Low-level laser therapy (LLLT)

#### Irradiation protocol

Laser irradiation was performed in a non-contact mode, with the tip positioned approximately 1 mm above the occlusal surface and oriented perpendicular to the long axis of the tooth. The irradiation distance was standardized using a rubber stopper adapted from an endodontic file, which was positioned laterally on the protective sleeve of the handpiece without obstructing or altering the laser beam path. This approach ensured consistent and reproducible positioning of the laser tip throughout all irradiation sessions.

Each treatment session consisted of two consecutive irradiation cycles of 30 s each, resulting in a total exposure time of 60 s per session. LLLT was administered on alternate days. Animals in the 1-week group received three irradiation sessions, whereas those in the 2- and 4-week groups received six sessions.

The occlusal irradiation approach was selected because it provided the most direct and reproducible access to the cavity preparation site in the rat maxillary first molars. Due to the limited oral cavity dimensions and restricted access to posterior teeth, alternative irradiation directions (e.g., buccal or lingual) would have compromised the ability to maintain a constant working distance, beam orientation, and reproducible positioning throughout the irradiation procedure. Positioning the laser perpendicular to the occlusal surface ensured standardized beam alignment across all specimens.

Previous studies have demonstrated that red-wavelength diode laser irradiation can penetrate dental hard tissues and transmit light energy to the underlying pulp, although transmission decreases with increasing tissue thickness and depends on the optical properties of the intervening tissues^33^. Experimental evidence has also shown that 650-nm laser irradiation can elicit photobiomodulatory responses in odontoblast-like cells^34^. Although PTFE and glass ionomer cement may partially attenuate light transmission, all specimens in the present study underwent identical restorative procedures and were irradiated using the same laser parameters, thereby ensuring standardized irradiation conditions across all experimental groups.

#### Laser parameters

Laser irradiation was delivered using a diode laser device (Woodpecker iLaser 116, Woodpecker^®^, China) equipped with a biostimulation handpiece. The beam diameter at the target tissue was approximately 8 mm, corresponding to a spot area of approximately 0.503 cm^2^. The beam diameter was estimated from the diameter of the biostimulation applicator used for irradiation and is consistent with published LLLT protocols employing the same Woodpecker I Laser 116 system under comparable irradiation conditions and with reports using an 8-mm LLLT applicator and the corresponding spot area calculation^27,35^. Because irradiation was performed in non-contact mode at a standardized working distance of approximately 1 mm, beam divergence over this short distance was considered negligible; therefore, the beam diameter at the target surface was considered to remain essentially unchanged. Wavelength: 650 nm.

- Output power: 100 mW.
- Operating mode: Continuous wave.
- Exposure time per session: 60 s.
- Energy delivered per session: 6 J.
- Beam diameter at target: approximately 8 mm.
- Spot area: approximately 0.503 cm^2^.
- Energy density (fluence): approximately 11.9 J/cm^2^.

#### Operational verification

The laser device was operated in accordance with the manufacturer’s instructions. According to the manufacturer, device calibration is performed using an external optical power meter and detector and is recommended annually through an authorized service facility. During the experimental period, the device remained within the manufacturer’s recommended calibration interval. Prior to each irradiation session, the preset treatment parameters (wavelength, output power, exposure time, and emission mode) were verified using the device settings, laser emission was confirmed using the device’s operational indicators, and the handpiece and biostimulation tip were inspected before each use to ensure proper function and integrity. These verification procedures were performed before every treatment session to help ensure consistent and reproducible laser delivery throughout the experimental period.

### Sample collection and histological processing

**Fig. 1:**
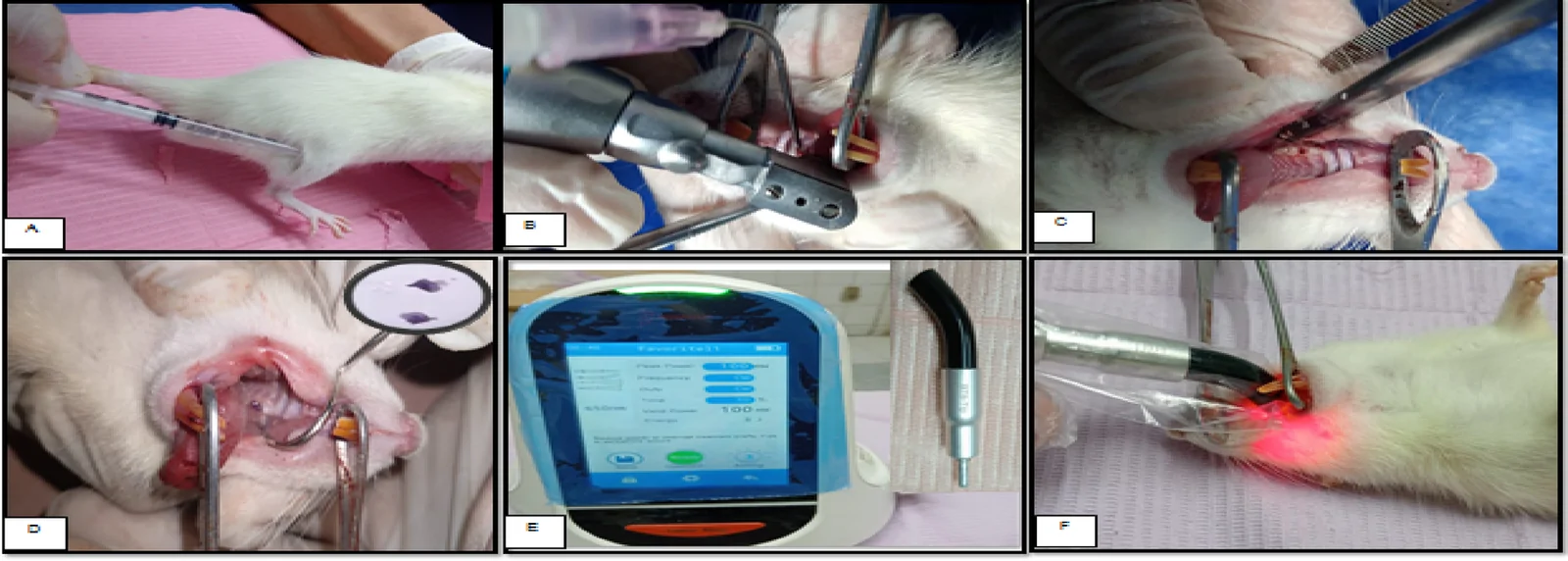
Direct pulp capping procedure and low-level laser therapy (LLLT) protocol in the rat molar model. (**a**) Induction of general anesthesia. (**b**) Standardized occlusal cavity preparation under continuous irrigation. (**c**) Mechanical pulp exposure. (**d**) Application of melatonin as a capping material to the exposed pulp. (**e**) LLLT device showing the laser tip configuration and irradiation parameters used during exposure cycles. (**f**) Laser irradiation applied to the occlusal surface of the molar.

At the designated time points (1, 2, and 4 weeks), animals were euthanized by anesthetic overdose in accordance with AVMA guidelines. Maxillary segments containing the first molars were dissected. The treated teeth were harvested and fixed in 10% neutral buffered formalin for 48 h, followed by decalcification in 10% EDTA (pH 7.4) for 4–6 weeks, as previously described .Specimens were then processed, embedded in paraffin, sectioned at 5 μm thickness, and stained with hematoxylin–eosin (H&E) and Masson’s trichrome (Fig. 1)^21^.

### Histological evaluation

Histological assessment was performed using criteria adapted from Guerrero-Girónes et al.^36^ and Sedek et al.^37^. The evaluated parameters included inflammatory cell infiltration (graded 0–4), pulp necrosis (absent/present), odontoblast-like layer organization (regular, irregular, or absent), fibrosis (absent/present), and vascularity (semi-quantitatively assessed) (Table 1). Dentin bridge thickness was measured on calibrated digital photomicrographs using ImageJ software (version 1.53 m; National Institutes of Health, Bethesda, MD, USA). Images were calibrated using the microscope scale bar before analysis. Three linear measurements were obtained perpendicular to the dentin bridge (one central and two equidistant peripheral measurements), and the mean value was recorded for each specimen. Reparative dentin formation was then scored according to the modified criteria, which incorporated dentin bridge thickness into the original histological assessment, with a mean dentin bridge thickness of ≥ 100 μm considered indicative of reparative dentin formation, based on the threshold proposed by Sedek et al.^38^. All sections were independently evaluated by two blinded examiners, and discrepancies were resolved by consensus.

**Table 1:** Histological scoring criteria used in the present study. (adapted from Guerrero-Girónes et al.^36^ and modified according to Sedek et al.^37^)

Criterion	Score	Description
Pulp inflammation	0	Absence of inflammation
	1	Mild inflammation
	2	Moderate inflammation
	3	Severe inflammation
	4	Abscess
Pulp necrosis	0	Absence
	1	Presence
Dentinal bridge and reparative dentin formation	0	Absence Mean dentin bridge thickness < 100 μm
	1	Presence Mean dentin bridge thickness ≥ 100 μm (modified from Sedek et al.^37^)
Odontoblast-like layer	0	Absence
	1	Irregular
	2	Regular
Fibrotic tissue	0	Absence
	1	Presence
Vascularity	1	Mild vascularity (few blood vessels)
	2	Moderate vascularity (moderate number of blood vessels)
	3	Severe vascularity (numerous blood vessels)

### Statistical analysis

Statistical analyses were performed using IBM SPSS Statistics for Windows, Version 27.0 (IBM Corp., Armonk, NY, USA). Normality of dentin bridge thickness data was assessed using the Shapiro–Wilk test. Dentin bridge thickness is presented as mean ± standard deviation (SD). Comparisons among the four study groups at each evaluation period (Weeks 1, 2, and 4) were performed using one-way analysis of variance (ANOVA), followed by Tukey’s post hoc test for multiple comparisons.

Histological parameters (inflammation, vascularity, necrosis, fibrosis, odontoblast-like layer organization, and dentin bridge formation) are presented as median and interquartile range (IQR). Comparisons among the study groups at each evaluation period were performed using the Kruskal–Wallis test, followed by Dunn’s post hoc test for pairwise comparisons when appropriate. To evaluate the predefined effect of adjunctive low-level laser therapy (LLLT), planned pairwise comparisons were performed using the Mann–Whitney U test between Group I (PTFE) and Group III (PTFE + LLLT), and between Group II (MEL) and Group IV (MEL + LLLT) at each evaluation period. All tests were two-tailed, and *p* < 0.05 was considered statistically significant.

## Results

### Negative control (normal pulp tissue)

**Fig. 2:**
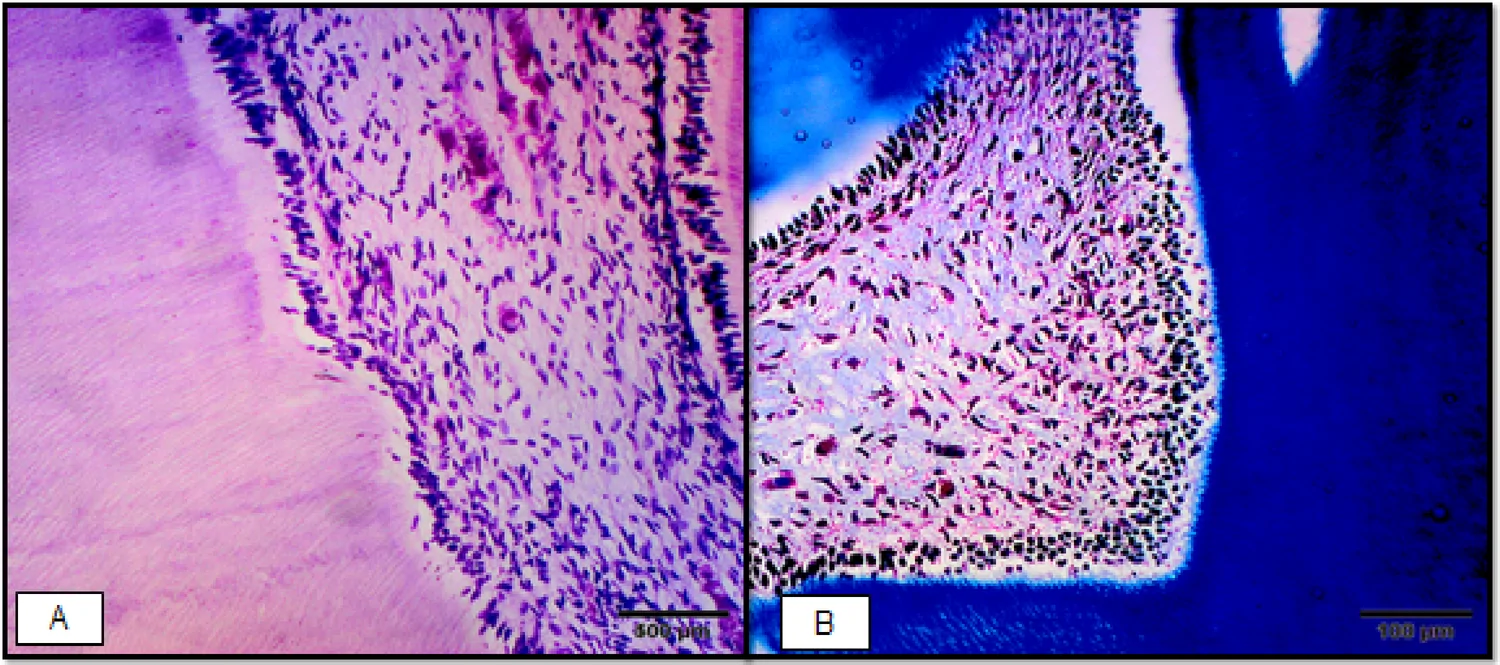
Representative photomicrographs of normal pulp tissue (negative control group). (**A**) Hematoxylin and eosin (H&E)–stained section showing intact odontoblast layer, organized connective tissue, normal vascular structures, and absence of inflammation. (**B**) Masson’s trichrome–stained section demonstrating regularly arranged collagen fibers and preserved extracellular matrix architecture.

Histological examination of the negative control specimens revealed normal pulpal architecture with no evidence of inflammatory or degenerative changes. In hematoxylin and eosin (H&E) and Masson’s trichrome–stained sections, the pulp tissue exhibited an intact and continuous odontoblast layer lining the dentin wall, well-organized connective tissue stroma, normal vascular structures, and absence of inflammatory cell infiltration. Fine, regularly arranged collagen fibers distributed uniformly within the pulp core, confirming preserved extracellular matrix organization and structural integrity (Fig. 2A, B). These findings served as the baseline reference for comparison with the experimentally treated groups.

### Inflammation

**Fig. 3:**
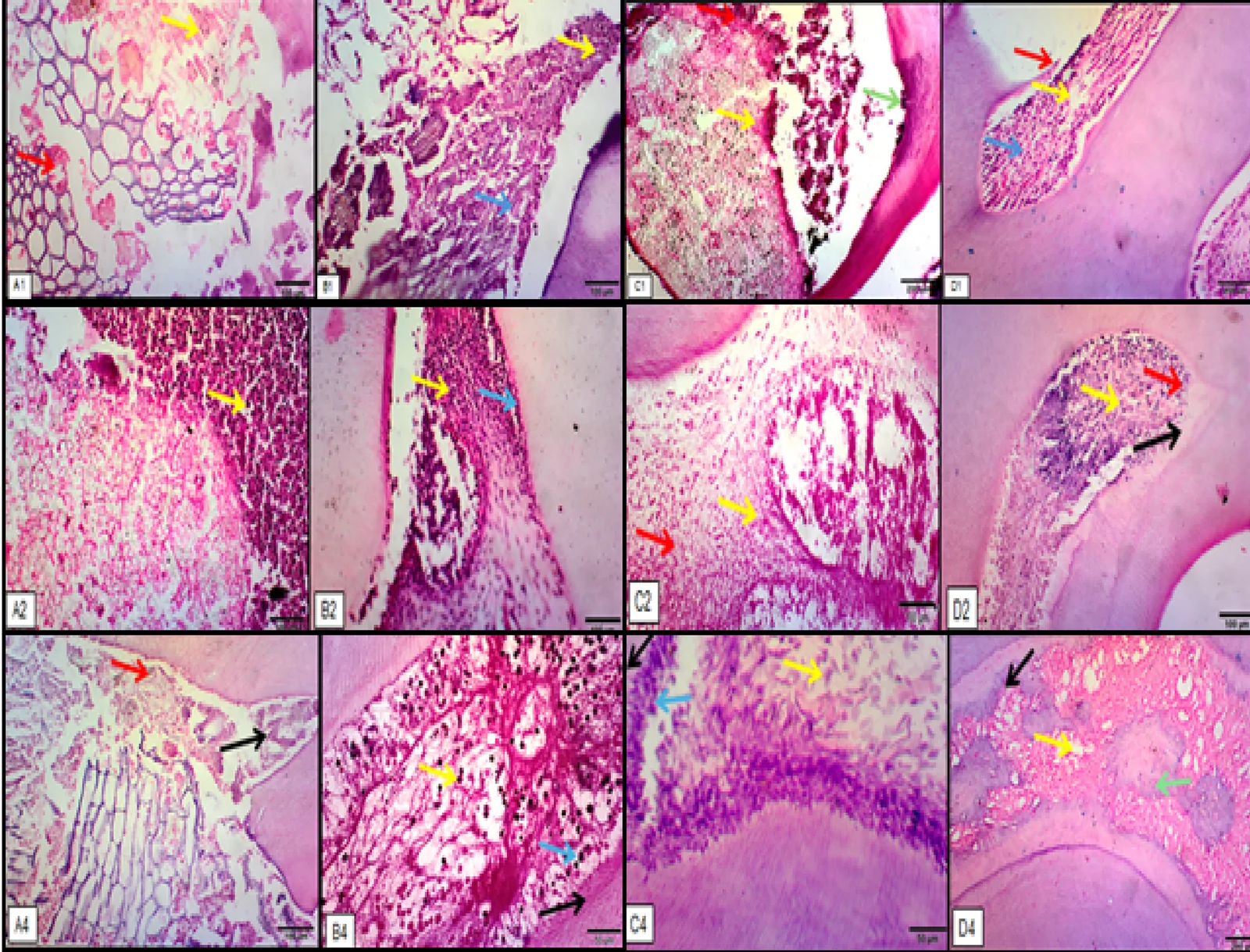
Representative hematoxylin and eosin (H&E)-stained photomicrographs of the treatment groups: Group I (PTFE), Group II (MEL), Group III (PTFE + LLLT), and Group IV (MEL + LLLT) at Weeks 1, 2, and 4. Scale bars = 50 μm. Week 1: (**A1**) Group I (PTFE): intense inflammatory infiltrate (yellow arrow), early degenerative changes, and vascular congestion (red arrowhead). (**B1**) Group II (MEL): reduced inflammation (yellow arrow), partial preservation of pulp architecture, and early fibroblastic proliferation (blue arrowhead). (**C1**) Group III (PTFE + LLLT): moderate inflammatory infiltrate (yellow arrow), initial connective tissue organization (red arrow), and early reparative activity at the pulp–dentin interface (green arrowhead). (**D1**) Group IV (MEL + LLLT): minimal inflammation (yellow arrow), organized pulp tissue with aligned fibroblasts (blue arrowhead), and an early odontoblast-like layer along the predentin (red arrow), indicating accelerated repair. **Week 2**: (**A2**) Group I (PTFE): persistent inflammatory infiltrate in the coronal pulp (yellow arrow). (**B2**) Group II (MEL): organized odontoblast-like cell layer (blue arrow) with minimal residual inflammation (yellow arrow). (**C2**) Group III (PTFE + LLLT): reduced inflammatory cell infiltration (yellow arrow) and improved pulpal organization (red arrow). (**D2**) Group IV (MEL + LLLT): continuous dentin bridge formation (black arrow), aligned odontoblast-like cells (red arrow), and normal pulp architecture (yellow arrow), indicating advanced healing. Week 4: (**A4**) Group I (PTFE): incomplete dentin bridge formation (black arrow) with mild residual inflammation (red arrow). (**B4**) Group II (MEL): irregular dentin bridge formation (black arrow), a distinct odontoblast-like cell layer (blue arrow), and minimal residual inflammation (yellow arrow). (**C4**) Group III (PTFE + LLLT): mineralized reparative dentin (black arrow), aligned odontoblast-like cells (blue arrow), and markedly reduced inflammation (yellow arrow). (**D4**) Group IV (MEL + LLLT): continuous tubular dentin bridge (black arrow) bordered by polarized odontoblast-like cells, fully organized inflammation-free pulp tissue, free calcific nodules (green arrow), and increased vascularity (yellow arrow), consistent with advanced pulpal healing.

**Fig. 4:**
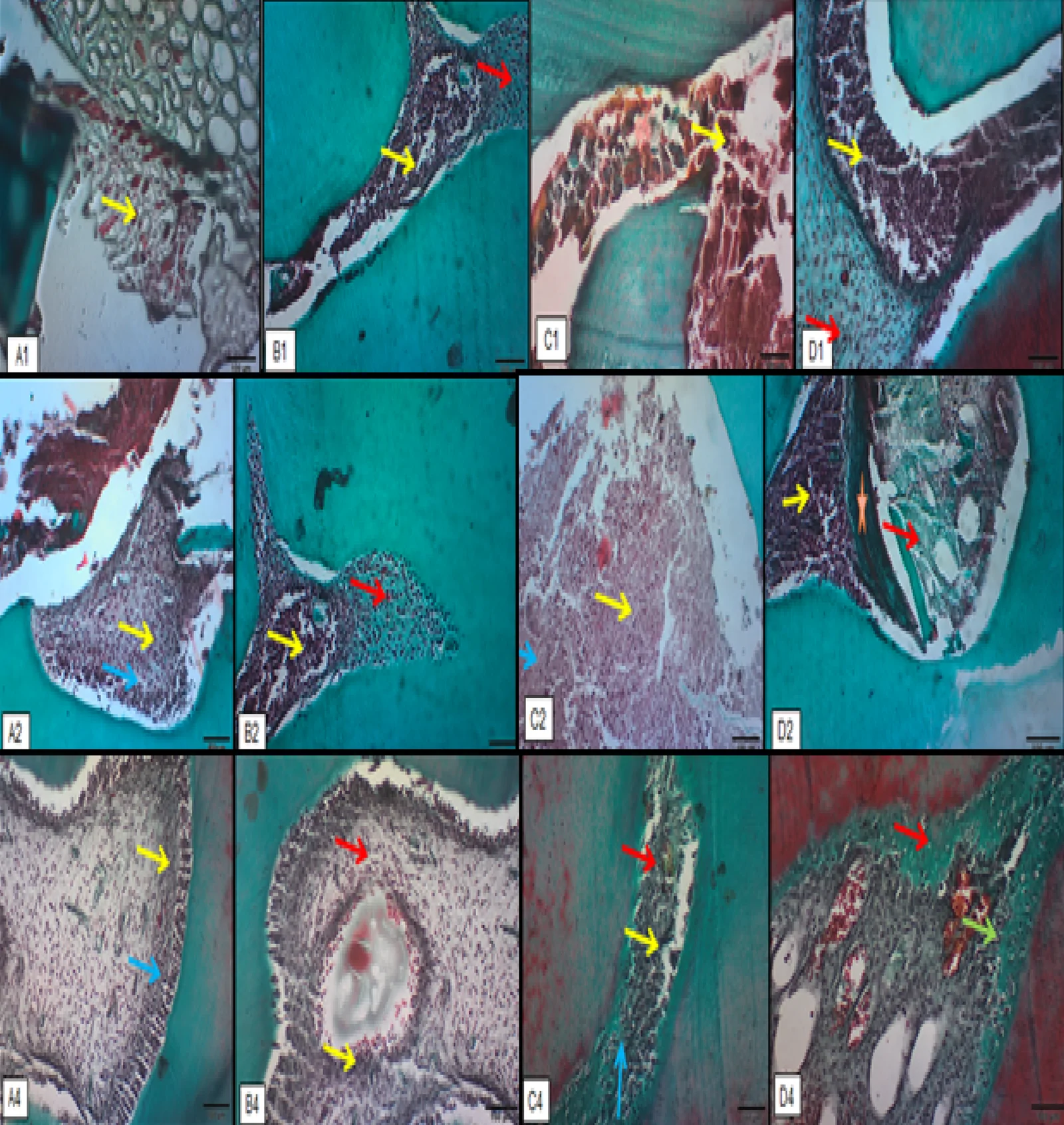
Representative Masson’s trichrome-stained photomicrographs of the treatment groups: Group I (PTFE), Group II (MEL), Group III (PTFE + LLLT), and Group IV (MEL + LLLT) at Weeks 1, 2, and 4. Scale bar = 50 μm. **Week 1**: (**A1**) Group I (PTFE): dense inflammatory infiltrate (yellow arrow). (**B1**) Group II (MEL): reduced inflammation (yellow arrow) with early connective tissue organization (red arrow). (**C1**) Group III (PTFE + LLLT): moderate inflammatory response (yellow arrow). (**D1**) Group IV (MEL + LLLT): minimal inflammation (yellow arrow), organized collagen matrix, and early fibroblast alignment (red arrow). **Week 2**: (**A2**) Group I (PTFE): persistent inflammation (yellow arrow) with irregular collagen deposition beneath the exposure site (blue arrowhead). (**B2**) Group II (MEL): reduced inflammation (yellow arrow) and early collagen fiber organization within the reparative zone (red arrow). (**C2**) Group III (PTFE + LLLT): moderate inflammation (yellow arrow) with developing collagen framework and focal mineralized matrix deposition (blue arrowhead). (**D2**) Group IV (MEL + LLLT): marked tissue organization with minimal inflammation (yellow arrow), aligned collagen bundles (red arrow), early odontoblast-like cell arrangement along the pulpal wall, and calcific bridge formation (star). **Week 4**: (**A4**) Group I (PTFE): persistent fibrosis with dense collagen bundles (blue arrowhead) and residual inflammatory cells (yellow arrow). (**B4**) Group II (MEL): moderate tissue organization with partial collagen maturation (red arrow) and reduced inflammation (yellow arrow). (**C4**) Group III (PTFE + LLLT): organized connective tissue (red arrow) with fewer inflammatory cells (yellow arrow), although uneven collagen distribution persists (blue arrowhead). (**D4**) Group IV (MEL + LLLT): complete tissue remodeling with a continuous, well-organized collagen matrix (red arrow), aligned fibroblasts, and a well-defined odontoblast-like layer along the dentin wall (green arrowhead), indicating advanced reparative dentin formation.

Inflammation scores differed significantly among the study groups at Weeks 1, 2, and 4 (Kruskal–Wallis test; *p* = 0.008, 0.008, and 0.007, respectively; Table 2). Group IV (MEL + LLLT) exhibited the lowest median inflammation scores at all evaluation periods. Post hoc analysis showed significantly lower inflammation scores in Group IV (MEL + LLLT) than in Group I (PTFE) at all evaluation periods and in Group III (PTFE + LLLT) at Weeks 2 and 4. Planned pairwise comparisons (Table 5), performed to evaluate the predefined effect of adjunctive LLLT within each pulp-capping material, showed significantly lower inflammation scores in Group III (PTFE + LLLT) than in Group I (PTFE) at Week 1 (*p* = 0.040) and in Group IV (MEL + LLLT) than in Group II (MEL) at Weeks 2 and 4 (*p* = 0.015 and 0.013, respectively). These quantitative findings were consistent with the histological observations, which showed a progressive decrease in inflammatory cell infiltration over time, particularly in specimens from Group IV (Figs. 3 and 4).

### Necrosis

Necrosis scores did not differ significantly among the study groups at Weeks 1, 2, or 4 (Kruskal–Wallis test; *p* = 0.061, 0.238, and 0.392, respectively; Table 3. Groups II (MEL) and IV (MEL + LLLT) consistently exhibited median necrosis scores of 0.00 throughout the experimental period, whereas Groups I (PTFE) and III (PTFE + LLLT) exhibited low necrosis scores at Weeks 1 and 2, which decreased to a median value of 0.00 at Week 4. Planned pairwise comparisons (Table 5), performed to assess the effect of adjunctive LLLT within each treatment modality, revealed no significant differences between Groups I and III or between Groups II and IV at any evaluation period (*p* > 0.05). Histologically, early degenerative changes were observed primarily in Group I at Week 1 (Fig. 3A1), whereas the remaining groups generally exhibited preserved pulpal architecture. By Weeks 2 and 4, histological sections showed more organized pulpal tissue with little to no evidence of necrotic changes across all groups (Figs. 3 and 4).

### Odontoblast-like layer

Odontoblast-like layer scores showed no significant differences among the study groups at Week 1 (*p* = 0.139), whereas significant differences were observed at Weeks 2 and 4 (*p* = 0.014 and 0.013, respectively; Table 3). Group IV (MEL + LLLT) exhibited the highest median scores at both time points. Post hoc analysis revealed significantly higher odontoblast-like layer scores in Group IV (MEL + LLLT) than in Groups I (PTFE) and II (MEL) at both Weeks 2 and 4. Planned pairwise comparisons (Table 5), performed to evaluate the predefined effect of adjunctive LLLT within each treatment modality, demonstrated significantly higher odontoblast-like layer scores in Group IV (MEL + LLLT) than in Group II (MEL) at Weeks 2 and 4 (*p* = 0.011 for both comparisons), whereas no significant differences were detected between Groups I (PTFE) and III (PTFE + LLLT). Histologically, odontoblast-like cells became progressively more organized over time, ranging from early alignment along the predentin border at Week 1 to a well-defined polarized odontoblast-like layer at Weeks 2 and 4, with the greatest degree of odontoblast-like layer organization observed in Group IV (Figs. 3 and 4).

### Reparative dentin (dentin bridge formation)

Dentin bridge formation scores did not differ significantly among the study groups at Weeks 1, 2, or 4 (*p* = 1.000, 0.172, and 0.475, respectively; Table 4). No dentin bridge formation was observed in any group at Week 1. At Week 2, Group IV (MEL + LLLT) exhibited the highest median score, whereas Groups I–III showed limited or early dentin bridge formation. By Week 4, dentin bridge formation was observed in all treatment groups, with the highest median scores recorded in Group IV. Planned pairwise comparisons (Table 5), performed to assess the effect of adjunctive LLLT within each treatment modality, showed no significant differences between Groups I and III or between Groups II and IV at any evaluation period (*p* > 0.05). Histologically, reparative dentin formation progressed from absent or minimal deposition at Week 1 to evident bridge formation at later time points, with Group IV exhibiting a continuous dentin bridge associated with organized odontoblast-like cells and histological features consistent with more advanced reparative dentin formation, whereas Groups I–III showed less consistent and less organized reparative dentin formation (Figs. 3 and 4).

### Dentin bridge thickness

Dentin bridge thickness increased progressively over the experimental period in all study groups (Table 4). At Week 1, the mean dentin bridge thickness was 4.36 ± 2.67 μm in Group I, 15.92 ± 4.12 μm in Group II, 20.01 ± 3.59 μm in Group III, and 25.33 ± 3.81 μm in Group IV. At Week 2, the corresponding values increased to 42.16 ± 1.46 μm in Group I, 94.29 ± 5.14 μm in Group II, 104.92 ± 6.88 μm in Group III, and 117.49 ± 13.24 μm in Group IV. By Week 4, the mean dentin bridge thickness reached 101.28 ± 1.98 μm in Group I, 146.46 ± 33.30 μm in Group II, 154.43 ± 42.64 μm in Group III, and 208.33 ± 1.54 μm in Group IV. One-way ANOVA demonstrated significant intergroup differences at each evaluation period (all *p* < 0.001). Post hoc Tukey’s test showed that Group IV exhibited significantly greater dentin bridge thickness than Group I at all evaluation periods, whereas the significance of the remaining pairwise comparisons varied across the evaluation periods (Table 4).

### Fibrosis

Fibrosis scores did not differ significantly among the study groups at Weeks 1, 2, or 4 (*p* = 0.599, 0.159, and 0.093, respectively; Table 3). Planned pairwise comparisons (Table 5), performed to assess the effect of adjunctive LLLT within each treatment modality, revealed no significant differences between Groups I and III or between Groups II and IV at any evaluation period (*p* > 0.05). Histologically, collagen deposition and connective tissue organization were observed in all groups and appeared more organized at later evaluation periods, with gradual maturation of fibrous tissue architecture over time (Figs. 3 and 4).

### Vascularity

**Fig. 5:**
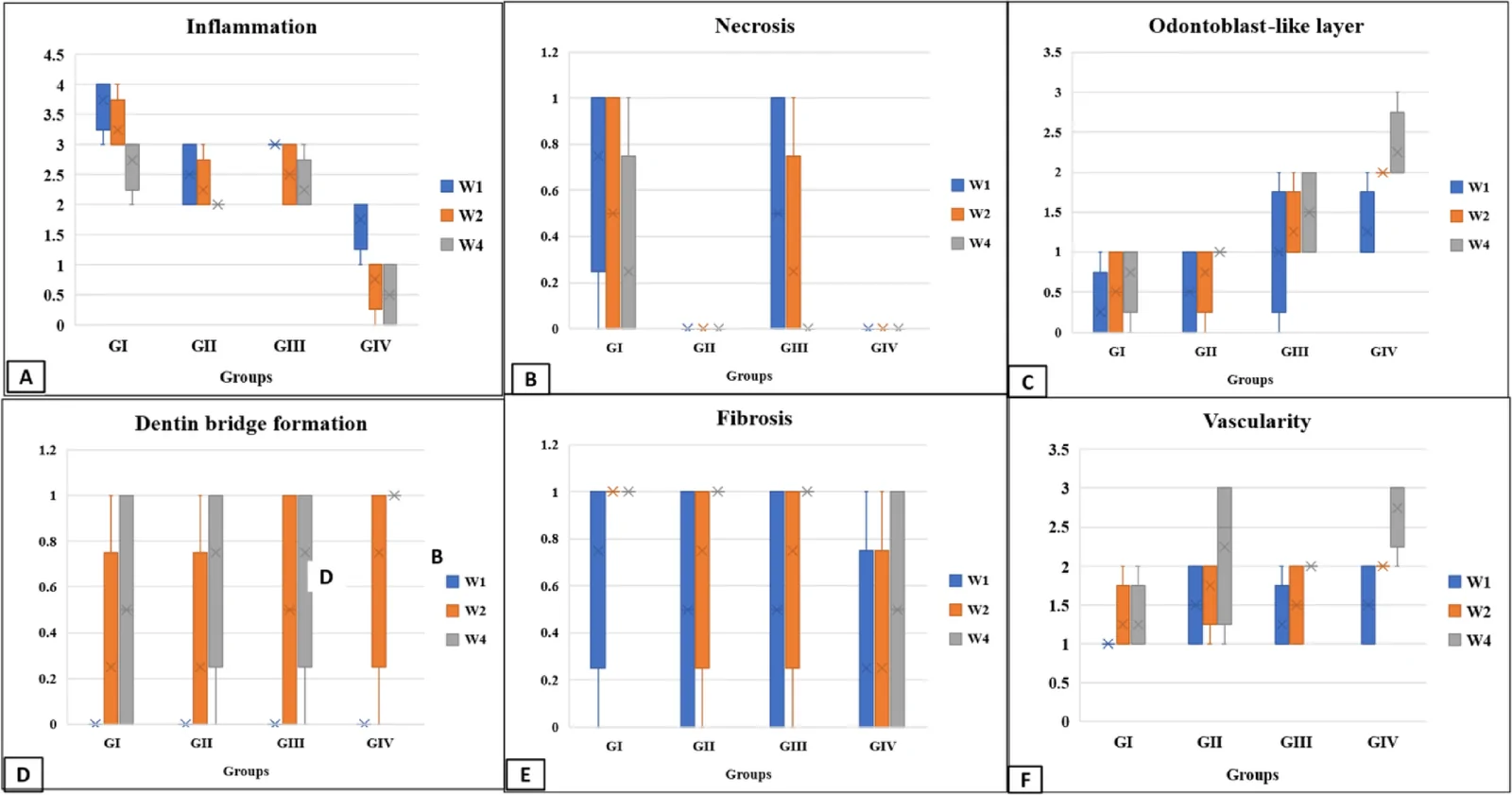
Histogram for scoring system for different treatment groups at Weeks 1, 2, and 4. (**A**) Inflammatory cell infiltration. (**B**) Pulp necrosis. (**C**) Odontoblast-like cell organization. (**D**) Dentin bridge formation. (**E**) Fibrotic tissue. (**F**) Vascular changes.

Vascularity scores did not differ significantly among the study groups at Weeks 1 and 2 (*p* = 0.392 and 0.172, respectively), whereas a significant intergroup difference was observed at Week 4 (*p* = 0.049; Table 2). Group IV (MEL + LLLT) exhibited the highest median vascularity scores, particularly at Week 4. Planned pairwise comparisons (Table 5), performed to evaluate the effect of adjunctive LLLT within each treatment modality, demonstrated significantly higher vascularity scores in Group III (PTFE + LLLT) than in Group I (PTFE) at Week 4 (*p* = 0.040), whereas no significant differences were observed between Groups II (MEL) and IV (MEL + LLLT) at any evaluation period (*p* > 0.05). Histologically, vascular structures were observed in all groups at all time points, with more evident vascular organization at later evaluation periods, particularly in Group IV at Week 4, alongside more organized tissue architecture (Figs. 3, 4, 5).

**Table 2:** Inflammation and vascularity scores in all study groups at different evaluation periods (1, 2, and 4 weeks).

Group	1 week	2 weeks	4 weeks
	Median (IQR)	Median (IQR)	Median (IQR)
Inflammation score			
Group I	4.00 (3.25–4.00)ᵃ	3.00 (3.00–3.75)ᵃ	3.00 (2.25–3.00)ᵃ
Group II	2.50 (2.00–3.00)ᵇᶜ	2.00 (2.00–2.75)ᵃᵇ	2.00 (2.00–2.00)ᵃᵇ
Group III	3.00 (3.00–3.00)ᵃᵇ	2.50 (2.00–3.00)ᵃ	2.00 (2.00–2.75)ᵃ
Group IV	2.00 (1.25–2.00)ᶜ	1.00 (0.25–1.00)ᵇ	0.50 (0.00–1.00)ᵇ
H	11.925	11.85	12.021
P-value	0.008*	0.008*	0.007*
Vascularity			
Group I	1.00 (1.00–1.00)	1.00 (1.00–1.75)	1.00 (1.00–1.75)ᵃ
Group II	1.50 (1.00–2.00)	2.00 (1.25–2.00)	2.50 (1.25–3.00)ᵃᵇ
Group III	1.00 (1.00–1.75)	1.50 (1.00–2.00)	2.00 (2.00–2.00)ᵃᵇ
Group IV	1.50 (1.00–2.00)	2.00 (2.00–2.00)	3.00 (2.25–3.00)ᵇ
H	3.00	5.00	7.86
P-value	0.392	0.172	0.049*

Values are presented as median (interquartile range [IQR]). H = Kruskal–Wallis test statistic. Different superscript lowercase letters within the same column indicate statistically significant differences according to Dunn’s post hoc multiple-comparison test. *P < 0.05.

**Table 3:** Necrosis, fibrosis, and odontoblast-like layer scores in all study groups at different evaluation periods (1, 2, and 4 weeks).

Group	1 week	2 weeks	4 weeks
	Median (IQR)	Median (IQR)	Median (IQR)
Necrosis score			
Group I	1.00 (0.25–1.00)	0.50 (0.00–1.00)	0.00 (0.00–0.75)
Group II	0.00 (0.00–0.00)	0.00 (0.00–0.00)	0.00 (0.00–0.00)
Group III	0.50 (0.00–1.00)	0.00 (0.00–0.75)	0.00 (0.00–0.00)
Group IV	0.00 (0.00–0.00)	0.00 (0.00–0.00)	0.00 (0.00–0.00)
H	7.364	4.231	3.00
P-value	0.061	0.238	0.392
Fibrosis			
Group I	1.00 (0.25–1.00)	1.00 (1.00–1.00)	1.00 (1.00–1.00)
Group II	0.50 (0.00–1.00)	1.00 (0.25–1.00)	1.00 (1.00–1.00)
Group III	0.50 (0.00–1.00)	1.00 (0.25–1.00)	1.00 (1.00–1.00)
Group IV	0.00 (0.00–0.75)	0.00 (0.00–0.75)	0.50 (0.00–1.00)
H	1.875	5.182	6.429
P-value	0.599	0.159	0.093
Odontoblast-like layer			
Group I	0.00 (0.00–0.75)	0.50 (0.00–1.00)^b^	1.00 (0.25–1.00)^a^
Group II	0.50 (0.00–1.00)	1.00 (0.25–1.00)^b^	1.00 (1.00–1.00)^a^
Group III	1.00 (0.25–1.75)	1.00 (1.00–1.75)^ab^	1.50 (1.00–2.00)^ab^
Group IV	1.00 (1.00–1.75)	2.00 (2.00–2.00)^a^	2.00 (2.00–2.75)^b^
H	5.491	10.562	10.861
P-value	0.139	0.014*	0.013*

Values are presented as median (interquartile range [IQR]). H = Kruskal–Wallis test statistic. Different superscript lowercase letters within the same column indicate statistically significant differences according to Dunn’s post hoc multiple-comparison test. *P < 0.05.

**Table 4:** Dentin bridge thickness and dentin bridge formation scores in all study groups at different evaluation periods (1, 2, and 4 weeks).

Group	Dentin bridge thickness (µm)		
	1 week	2 weeks	4 weeks
	Mean ± SD	Mean ± SD	Mean ± SD
Group I	4.36 ± 2.67^c^	42.16 ± 1.46^c^	101.28 ± 1.98^b^
Group II	15.92 ± 4.12^b^	94.29 ± 5.14^b^	146.46 ± 33.30^b^
Group III	20.01 ± 3.59^ab^	104.92 ± 6.88^ab^	154.43 ± 42.64^ab^
Group IV	25.33 ± 3.81^a^	117.49 ± 13.24^a^	208.33 ± 1.54^a^
F	24.63	69.75	10.51
P-value	< 0.001**	< 0.001**	< 0.001**

Group	Dentin bridge formation		
	Median (IQR)	Median (IQR)	Median (IQR)
Group I	0.00 (0.00–0.00)	0.00 (0.00–0.00)	0.50 (0.00–1.00)
Group II	0.00 (0.00–0.00)	0.00 (0.00–0.75)	1.00 (0.25–1.00)
Group III	0.00 (0.00–0.00)	0.50 (0.00–1.00)	1.00 (0.25–1.00)
Group IV	0.00 (0.00–0.00)	1.00 (0.25–1.00)	1.00 (1.00–1.00)
H	0.00	5.00	2.50
P-value	1.00	0.172	0.475

Values are presented as mean ± SD or median (interquartile range [IQR]). F = ANOVA F-statistic; H = Kruskal–Wallis test statistic. Different superscript lowercase letters within the same column indicate statistically significant differences according to Dunn’s post hoc multiple-comparison test. *P < 0.05; **P < 0.001.

**Table 5:** Planned pairwise comparisons of histological parameters between Group I (PTFE) and Group III (PTFE + LLLT), and between Group II (MEL) and Group IV (MEL + LLLT) at different evaluation periods.

Parameter	Time	GI Median (IQR)	GIII Median (IQR)	U	*P*-value	GII Median (IQR)	GIV Median (IQR)	U	*P*-value
Inflammation	W1	4.00 (3.25–4.00)	3.00 (3.00–3.00)	2.00	0.040*	2.50 (2.00–3.00)	2.00 (1.25–2.00)	3.00	0.096
	W2	3.00 (3.00–3.75)	2.50 (2.00–3.00)	3.00	0.096	2.00 (2.00–2.75)	1.00 (0.25–1.00)	0.00	0.015*
	W4	3.00 (2.25–3.00)	2.00 (2.00–2.75)	4.00	0.186	2.00 (2.00–2.00)	0.50 (0.00–1.00)	0.00	0.013*
Vascularity	W1	1.00 (1.00–1.00)	1.00 (1.00–1.75)	6.00	0.317	1.50 (1.00–2.00)	1.50 (1.00–2.00)	8.00	1.000
	W2	1.00 (1.00–1.75)	1.50 (1.00–2.00)	6.00	0.495	2.00 (1.25–2.00)	2.00 (2.00–2.00)	6.00	0.317
	W4	1.00 (1.00–1.75)	2.00 (2.00–2.00)	2.00	0.040*	2.50 (1.25–3.00)	3.00 (2.25–3.00)	5.50	0.405
Necrosis	W1	1.00 (0.25–1.00)	0.50 (0.00–1.00)	6.00	0.495	0.00 (0.00–0.00)	0.00 (0.00–0.00)	8.00	1.000
	W2	0.50 (0.00–1.00)	0.00 (0.00–0.75)	6.00	0.495	0.00 (0.00–0.00)	0.00 (0.00–0.00)	8.00	1.000
	W4	0.00 (0.00–0.75)	0.00 (0.00–0.00)	6.00	0.317	0.00 (0.00–0.00)	0.00 (0.00–0.00)	8.00	1.000
Fibrosis	W1	1.00 (0.25–1.00)	0.50 (0.00–1.00)	6.00	0.495	0.50 (0.00–1.00)	0.00 (0.00–0.75)	6.00	0.495
	W2	1.00 (1.00–1.00)	1.00 (0.25–1.00)	6.00	0.317	1.00 (0.25–1.00)	0.00 (0.00–0.75)	4.00	0.186
	W4	1.00 (1.00–1.00)	1.00 (1.00–1.00)	8.00	1.000	1.00 (1.00–1.00)	0.50 (0.00–1.00)	4.00	0.127
Odontoblast-like layer	W1	0.00 (0.00–0.75)	1.00 (0.25–1.75)	3.50	0.155	0.50 (0.00–1.00)	1.00 (1.00–1.75)	3.00	0.096
	W2	0.50 (0.00–1.00)	1.00 (1.00–1.75)	3.00	0.096	1.00 (0.25–1.00)	2.00 (2.00–2.00)	0.00	0.011*
	W4	1.00 (0.25–1.00)	1.50 (1.00–2.00)	3.00	0.096	1.00 (1.00–1.00)	2.00 (2.00–2.75)	0.00	0.011*
Dentin bridge	W1	0.00 (0.00–0.00)	0.00 (0.00–0.00)	8.00	1.000	0.00 (0.00–0.00)	0.00 (0.00–0.00)	8.00	1.000
	W2	0.00 (0.00–0.75)	0.00 (0.00–0.00)	4.00	0.343	0.00 (0.00–0.75)	1.00 (0.25–1.00)	4.00	0.186
	W4	0.50 (0.00–1.00)	1.00 (0.25–1.00)	6.00	0.495	1.00 (0.25–1.00)	1.00 (1.00–1.00)	6.00	0.317

Data are presented as median (interquartile range [IQR]). Pairwise comparisons between groups were performed using the Mann–Whitney U test. U = Mann–Whitney test statistic. GI = PTFE; GII = MEL; GIII = PTFE + LLLT; GIV = MEL + LLLT; W1 = Week 1; W2 = Week 2; W4 = Week 4. *P < 0.05 indicates statistical significance.

## Discussion

Dental pulp capping is essential for preserving tooth vitality and avoiding invasive endodontic intervention^1,3,5^. In this rat molar direct pulp-capping model, adjunctive low-level laser therapy (LLLT) appeared to contribute to improved pulpal healing outcomes, while melatonin acted as a bioactive adjunct that may have optimized the local cellular microenvironment. Histological evaluation demonstrated distinct intergroup differences in inflammatory response, odontoblast-like layer organization, vascularity, and dentin bridge thickness. Necrosis, fibrosis, and dentin bridge formation scores showed more limited quantitative differences among groups.

The biological effects observed in the present study are consistent with previous evidence demonstrating that red-wavelength diode laser irradiation can penetrate dental hard tissues, although transmission decreases with increasing tissue thickness^33^. Experimental studies have shown that 650-nm diode laser irradiation upregulates VEGF-A and TGF-β1 expression in odontoblast-like cells, supporting its role in promoting angiogenesis and reparative dentinogenesis^34^. Although PTFE and glass ionomer cement may partially attenuate light transmission, favorable outcomes have been reported following laser-assisted vital pulp therapy despite the presence of restorative materials^38^. Collectively, these findings support the biological plausibility of the irradiation protocol used in the present study and provide a mechanistic basis for the improved inflammatory response, vascularity, odontoblast-like layer organization, and dentin bridge thickness observed in the laser-treated groups.

Inflammatory scores differed significantly among groups at all-time points, primarily driven by consistently lower values in Group IV (MEL + LLLT). Group IV (MEL + LLLT) exhibited the lowest values, with a significant decline over time, approaching near-complete resolution by Week 4. Group I (PTFE) showed persistent inflammation and early necrotic changes, consistent with its role as an inert experimental control lacking the bioactive properties of contemporary pulp-capping materials. Group II (MEL) displayed moderate improvement, though no significant intragroup temporal change was detected. Group III (PTFE + LLLT) showed progressive tissue organization, with non-significant intragroup trends. These findings align with LLLT’s mitochondrial photobiostimulation—interaction with cytochrome c oxidase, transient modulation of ROS and nitric oxide, and downstream suppression of NF-κB-dependent cytokine signaling^14,39^—and melatonin’s attenuation of oxidative stress and NF-κB activation in pulp-derived cells^40^. Together, these mechanisms foster a permissive microenvironment for early reparative events.

Necrosis scores did not differ significantly among the study groups at any evaluation period. Nevertheless, Groups I (PTFE) and III (PTFE + LLLT) exhibited low necrosis scores during the early healing phase, whereas Groups II (MEL) and IV (MEL + LLLT) consistently showed complete absence of necrosis throughout the study period. Although necrosis scores in Groups I and III decreased numerically over time, these changes were not statistically significant. The early and sustained absence of necrosis in Groups II and IV is biologically plausible given melatonin’s antioxidant and mitochondrial-stabilizing effects that limit oxidative injury and apoptosis in pulp-derived cells^17,40,41^. In Group IV, continued absence of necrosis likely reflects combined benefits of improved early inflammatory control and enhanced vascular support rather than a direct anti-necrotic effect. Notably, Group II also maintained full vitality throughout the observation period, suggesting a possible melatonin-associated protective effect independent of laser irradiation; formal mechanistic confirmation, however, was beyond the scope of this study^17,40,41^.

From Week 2 onward, the odontoblast-like layer showed a graded pattern of cellular organization. Group IV demonstrated a continuous, well-polarized odontoblast-like layer bordering newly formed predentin. Group III showed improved polarity and continuity, while Group II exhibited partial alignment, and Group I remained sparse and disorganized. These trends are consistent with previous reports that LLLT enhances odontogenic differentiation through increased expression of DSPP, DMP-1, and ALP, as well as activation of morphogenetic signaling pathways such as TGF-β1/Smad, which are critical for reparative dentinogenesis^34,42,43^. Melatonin further supports odontogenic differentiation via activation of MAPK signaling pathways (ERK/p38) and epigenetic modulation, including reduced DNMT1 and MeCP2 expression and decreased global DNA methylation, thereby facilitating mineralization-related gene expression in human dental pulp cells^17,40^.

Dentin bridge thickness increased progressively in all groups throughout the experimental period, with significant intergroup differences observed at all evaluation periods. Group IV consistently exhibited the greatest dentin bridge thickness, whereas Group I showed the lowest values. These findings are consistent with the proposed role of low-level laser therapy (LLLT) in promoting reparative dentinogenesis through stimulation of cellular activity and extracellular matrix deposition, while melatonin may enhance the local microenvironment by reducing oxidative stress and supporting odontogenic differentiation. These mechanisms have been attributed to laser-induced upregulation of growth factors involved in odontoblast differentiation and angiogenesis, together with the antioxidant and pro-odontogenic effects of melatonin on dental pulp cells^5,11,34,44^. The superior dentin bridge thickness observed in the combined-treatment group is further supported by the enhanced odontoblast-like cell organization and vascularity demonstrated histologically in the present study.

Although dentin bridge thickness differed significantly among groups at all time points, dentin bridge formation scores did not show statistically significant intergroup differences. This finding is consistent with the sequential nature of reparative dentinogenesis, whereby early cellular organization and extracellular matrix deposition precede the formation of a mature mineralized dentin bridge^12,44^.

Histological patterns included continuous tubular bridges in a subset of Group IV specimens, partial mineralization in Group III, irregular deposition in Group II, and sparse, disorganized mineralized areas in Group I.

Masson’s trichrome staining revealed denser, irregular bundles in Group I (disorganized repair), intermediate organization in Group II, and finer, more regularly aligned fibers in Groups III and IV, with the most organized architecture in Group IV. Despite these qualitative differences, fibrosis scores did not differ significantly among groups at any time point. LLLT has been associated with enhanced connective tissue remodeling by reducing inflammation and promoting organized collagen deposition in oral tissues^14,39,45^. Melatonin has been reported to exert antioxidant and anti-inflammatory effects that support tissue repair and modulation of the pulpal microenvironment^5,41^. Tissue specificity remains an important consideration, as collagen dynamics vary according to local cellular composition and microenvironment. In the present model, the more organized collagen architecture observed histologically in Group IV likely reflects improved early inflammatory regulation rather than a direct antifibrotic effect^14,45^.

Vascularity showed significant intergroup differences at Week 4, with the highest scores observed in Group IV (MEL + LLLT), whereas Groups II (MEL) and III (PTFE + LLLT) exhibited intermediate values and Group I (PTFE) showed the lowest scores. Histologically, Group I exhibited sparse and irregular capillary structures, while Group II showed moderate vascularization. Laser-treated groups (III and IV) showed a trend toward more organized vascular networks, with statistically significant differences emerging at Week 4, while Group IV demonstrated the most mature neovascular architecture. LLLT has been shown to upregulate angiogenic mediators, including vascular endothelial growth factor (VEGF) and TGF-β1, and to promote endothelial proliferation, thereby supporting pulp revascularization^34^. Clinical evidence further supports LLLT-mediated improvements in pulpal healing compared with conventional pulpotomy agents^46^.

Melatonin’s regenerative influence in dental pulp tissue has been attributed to its antioxidant and anti-inflammatory properties, which support wound healing and may indirectly enhance angiogenic responses within a reparative environment^41^. Specifically, melatonin has been shown to attenuate senescence in human dental pulp stem cells via inhibition of MMP3, modulating NF-κB and IL-6/JAK/STAT3 signaling pathways, thereby optimizing the local microenvironment for reparative processes^41^. Accordingly, the vascular patterns observed across groups are consistent with LLLT-driven angiogenesis occurring within a melatonin-optimized environment^7,34,41^.

Collectively, the present findings suggest that adjunctive LLLT, particularly when combined with melatonin, enhances pulpal healing through favorable effects on inflammation, vascularity, and odontoblast-like layer organization, while melatonin may contribute to a more favorable oxidative and inflammatory microenvironment. Whether the interaction is additive or synergistic cannot be determined from the present design^14,39,40^. Standardization of LLLT parameters—including wavelength, power output, energy density, beam profile, irradiation timing, and treatment frequency—is essential to ensure reproducibility and clinical translation^14,47^.

### Study limitations

The present study has several limitations. First, the experimental model was conducted in rats; therefore, the results cannot be directly applied to human clinical conditions without further validation through controlled clinical trials. Second, the relatively short follow-up period limited the assessment of the long-term stability and maturation of pulpal healing. Third, the pulpal healing assessment was based primarily on histological and histomorphometric evaluation, without complementary biochemical, molecular, or immunohistochemical analyses that could further explain the underlying healing mechanisms. In addition, quantitative histomorphometric measurements were limited to dentin bridge thickness. Future studies incorporating comprehensive quantitative image-analysis techniques, including inflammatory cell counts, vascular density, and collagen area fraction measurements, may provide a more detailed assessment of pulpal healing and strengthen the mechanistic interpretation of the findings.

## Conclusion

Within the limitations of this study, adjunctive low-level laser therapy (LLLT), particularly when combined with melatonin, was associated with improved histological pulpal healing outcomes, including reduced inflammation, enhanced vascularity, improved odontoblast-like layer organization, and increased dentin bridge thickness. Melatonin demonstrated supportive effects on the pulpal microenvironment and may have contributed to the overall healing response when used in combination with LLLT. Further studies are warranted to optimize LLLT parameters and elucidate the underlying biological mechanisms prior to clinical application in vital pulp therapy.

## Recommendation

It is strongly recommended that further experimental and clinical studies be conducted to validate and confirm these outcomes in teeth with different degrees of pulpal inflammation with longer observation periods.

## Data Availability

All data supporting the findings of this study are available within the paper and .the datasets used and analyzed during the current study are available from the corresponding author on reasonable request.
